# Supplementary material for: New capsaicin analogs as molecular rulers to define the permissive conformation of the mouse TRPV1 ligand-binding pocket
Source: eLife. 2020 Nov 9;9:e62039. doi: 10.7554/eLife.62039 (PMC7671684; doi:10.7554/eLife.62039)
Supplement: Supplementary file 1. — Rosetta commands and scripts for docking. [file elife-62039-supp1.docx]

Supplementary File 1

**(*E*)-*N*-(4-Hydroxy-3-methoxyphenethyl)-8-methylnon-6-enamide** (**Cap_+1_**): A suspension of 4-(2-aminoethyl)-2-methoxyphenol hydrochloride (**1)** (203 mg, 1.0 mM) in 8.0 mL chloroform and 4.0 mL THF was placed in a 20 mL flask and 300 μL (*E*)-8-methylnon-6-enoyl chloride (**2**) (285 mg, 1.5 mM) was added in a single portion. The suspension immediately turned into a purple brown solution. The mixture was stirred at room temperature (RT) for 6 h. It was then washed successively with saturated sodium bicarbonate solution and brine. The organic phase was dried over anhydrous sodium sulfate and the solvent was removed under reduced pressure to obtain 425 mg of a thick brown liquid which later solidified. The crude product was subjected to column chromatographic purification using cyclohexane and ethyl acetate as eluent, yielding 222 mg (70%) of **Cap_+1_** and 80 mg (55%) of **2-methoxy-4-(2-((*E*)-8-methylnon-6-enamido)ethyl)phenyl(*E*)-8-methylnon-6-enoate** as a byproduct.

**(*E*)-*N*-(4-Hydroxy-3-methoxyphenethyl)-8-methylnon-6-enamide** (**Cap_+1_**): White waxy solid M.p. 85.0-86.2 °C; ^1^H NMR (800 MHz, DMSO-d6): δ 8.72 (s, 1H), 7.82 (t, *J* = 5.6 Hz, 1H), 6.77 (d, *J* = 2.0 Hz, 1H), 6.70 (d, *J* = 7.9 Hz, 1H), 6.59 (dd, *J* = 7.9, 1.9 Hz, 1H), 5.41 – 5.39 (m, 1H), 5.36 – 5.33 (m, 1H), 3.77 (s, 3H), 3.25 – 3.23 (m, 2H), 2.61 (t, *J* = 7.4 Hz, 2H), 2.24 (h, *J* = 6.7 Hz, 1H), 2.06 (t, *J* = 7.4 Hz, 2H), 1.95 (q, *J* = 7.1 Hz, 2H), 1.49 (p, *J* = 7.5 Hz, 2H), 1.29 (p, *J* = 9.8, 7.5 Hz, 2H), 0.96 (d, *J* = 6.8 Hz, 6H). ^13^C NMR (200 MHz, DMSO-d6): *δ* = 22.5, 24.8, 28.6, 30.3, 31.6, 34.8, 35.2, 40.3, 55.4, 112.6, 115.2, 120.6, 126.6, 130.2, 137.3, 144.7, 147.3, 171.8.

**2-Methoxy-4-(2-((*E*)-8-methylnon-6-enamido)ethyl)phenyl(*E*)-8-methylnon-6-enoate**: White waxy solid M.p. 48.0-48.5 °C; ^1^H NMR (800 MHz, CDCl_3_): *δ* = 6.93 (d, *J* = 8.0 Hz, 1H), 6.77 (d, *J* = 1.60 Hz, 1H), 6.74 (dd, *J_1_* = 1.60 Hz*, J_2_* = 8.0 Hz, 1H), 5.52 (brs, 1 H), 5.41 - 5.29 (m, 4H), 3.79 (s, 3H), 3.50 (q, *J* = 6.50 Hz, 2H), 2.79 (t, *J* = 6.80 Hz, 2H), 2.57 (t, *J* = 7.50 Hz, 2H), 2.25 – 2.19 (m, 2H), 2.12 (t, *J* = 7.60 Hz, 2H), 2.03 (q, *J* = 7.20 Hz, 2H), 1.96 (q, *J* = 7.20 Hz, 2H), 1.76 (p, *J* = 7.60 Hz, 2H), 1.60 (p, *J* = 7.60 Hz, 2H), 1.49 (p, *J* = 7.60 Hz, 2H), 1.35 (p, *J* = 7.60 Hz, 2H), 0.958 (d, *J* = 12.8 Hz, 6H), 0.950 (d, *J* = 12.8 Hz, 6H). ^13^C NMR (200 MHz, CDCl_3_): *δ* = 22.84, 22.85, 24.7, 25.4, 29.1, 29.4, 31.15, 31.19, 32.3, 32.4, 34.0, 35.8, 36.8, 40.6, 56.0, 112.9, 120.9, 122.9, 126.66, 126.67, 137.9, 138.22, 138.28, 138.5, 151.2, 172.1, 173.3.

The structure of Cap_+1_ was further confirmed through a homo nuclear decoupling NMR experiment. Irradiation of the N-H proton of Cap_+1_ at *δ* = 7.8 (t, J = 5.7 Hz, 1H) led to a loss of its coupling with the neighboring -CH_2_ protons at *δ* = 3.23 – 3.17 (m, 2H) and the NMR signal of these -CH_2_ protons collapsed into a triplet from a multiplet. Reversely, irradiation of the -CH_2_ protons at *δ* = 3.23 – 3.17 (m, 2H) resulted in the loss of their coupling with the N-H proton at *δ* = 7.8 (t, J = 5.7 Hz, 1H) and the NMR signal of this N-H proton collapsed into a singlet from a triplet. As the irradiated -CH_2_ is adjacent to another set of benzylic -CH_2_ protons at *δ* = 2.57 (t, J = 7.4 Hz, 2H) the NMR signal of these -CH_2_ protons also collapsed into a singlet from a triplet. These results unambiguously proved that the nitrogen atom is bearing only one proton which is only possible if amine **(1)** was transformed into amide **(2)** during the reaction, thus confirming the structure of Cap_+1_. The structure of Cap_+1_ was further confirmed by single crystal x-ray diffraction (data not shown).

**(*E*)-*N*-(4-Hydroxy-3-methoxyphenyl)-8-methylnon-6-enamide** (**Cap_-1_**): A suspension of 4-amino-2-methoxyphenol (**3)** (280 mg, 2.0 mM) in 10.0 mL chloroform and 4.0 mL THF was placed in a 20 mL flask and 420 μL (*E*)-8-methylnon-6-enoyl chloride (**2**) (378 mg, 2.0 mM) was added in a single portion. The suspension immediately turned into a purple brown solution. The mixture was stirred at RT for 6 h. It was then washed successively with saturated sodium bicarbonate solution and brine. The organic phase was dried over anhydrous sodium sulfate and the solvent was removed under reduced pressure to obtain 600 mg of a thick brown liquid which later solidified. Crude product was subjected to column chromatographic purification using cyclohexane and ethyl acetate as eluent, yielding 451 mg (76%) of **Cap_-1_** as a white solid. M.p. 85.5-87.0 °C; ^1^H NMR (800 MHz, CDCl_3_): *δ* = 7.50 (s, 1H), 7.23 (brs, 1H), 6.82 (d, *J* = 8.4 Hz, 1H), 6.64 (dd, *J_1_* = 2.4, *J_2_* = 8.4 Hz, 1H), 5.40 – 5.37 (m, 1H), 5.34 – 5.31 (m, 1H), 3.87 (s, 3H), 2.33 (t, *J* = 7.6 Hz, 2H), 2.24 – 2.20 (m, 1H), 2.01 (q, *J* = 7.2 Hz, 2H), 1.71 (p, *J* = 7.68 Hz, 2H), 1.42 (p, *J* = 7.6 Hz, 2H), 0.95 (d, *J* = 6.8 Hz, 6H). ^13^C NMR (200 MHz, CDCl_3_): *δ* = 22.8, 25.2, 29.4, 31.1, 32.4, 37.7, 56.1, 104.6, 112.5, 114.2, 126.6, 131.0, 138.3, 142.5, 146.5, 171.4.


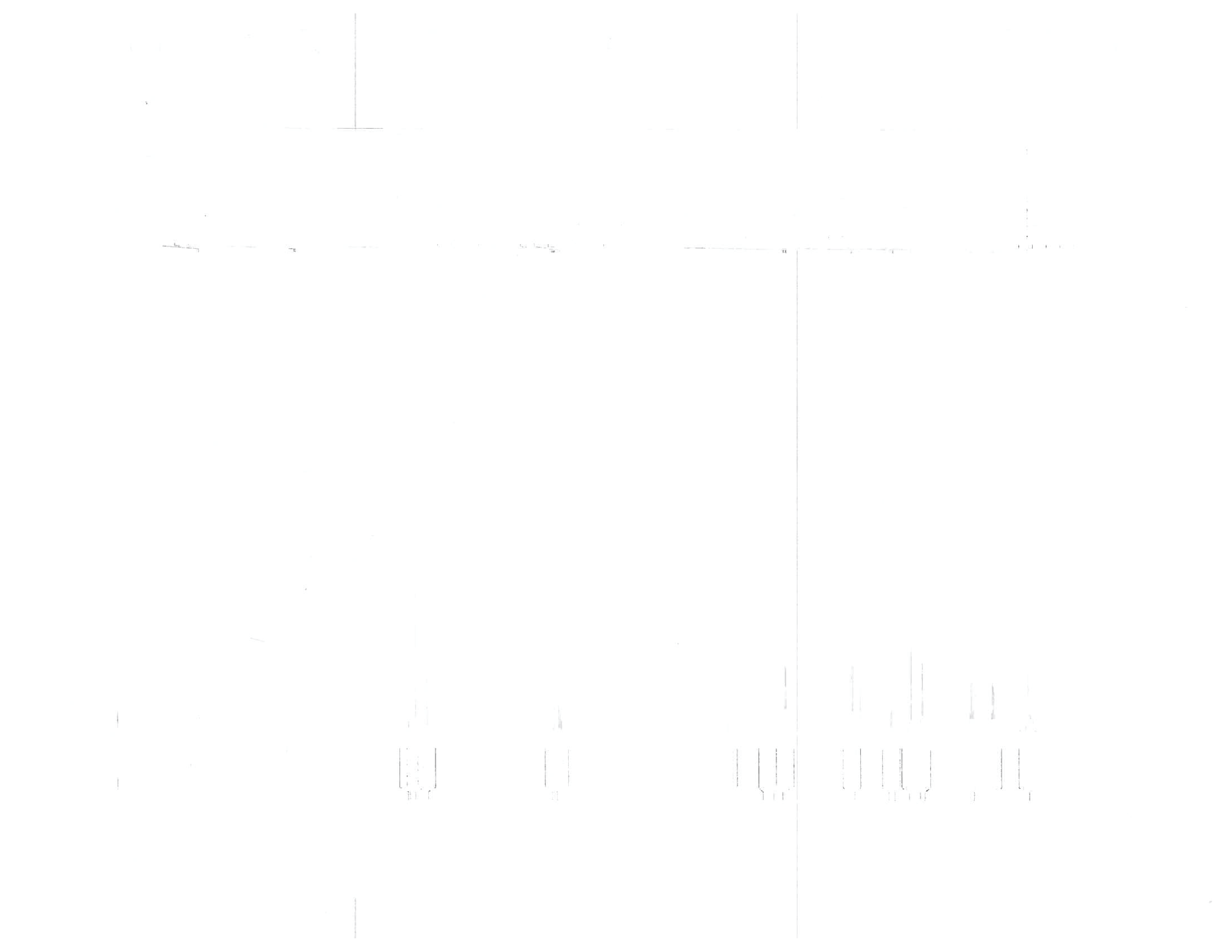

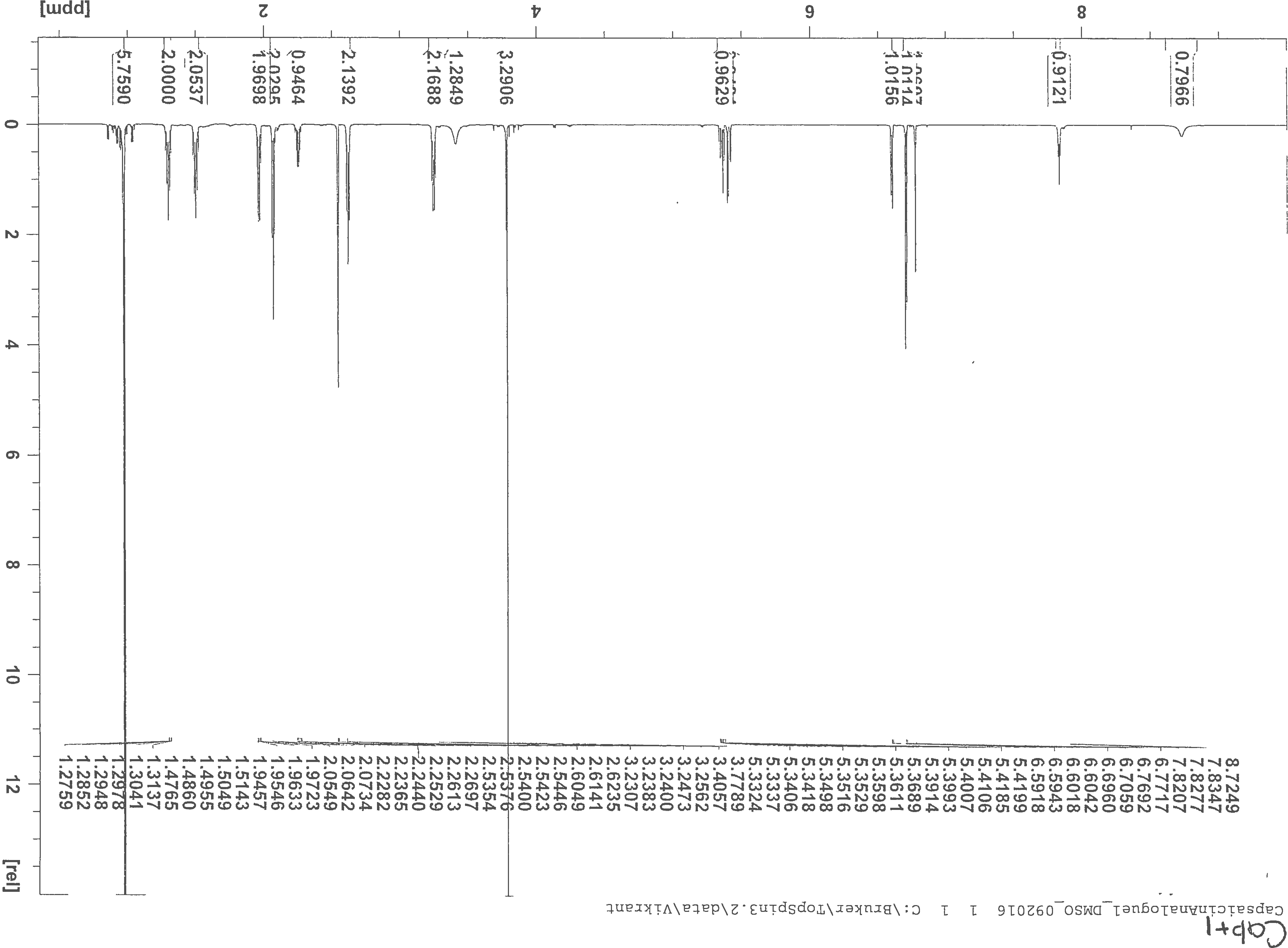

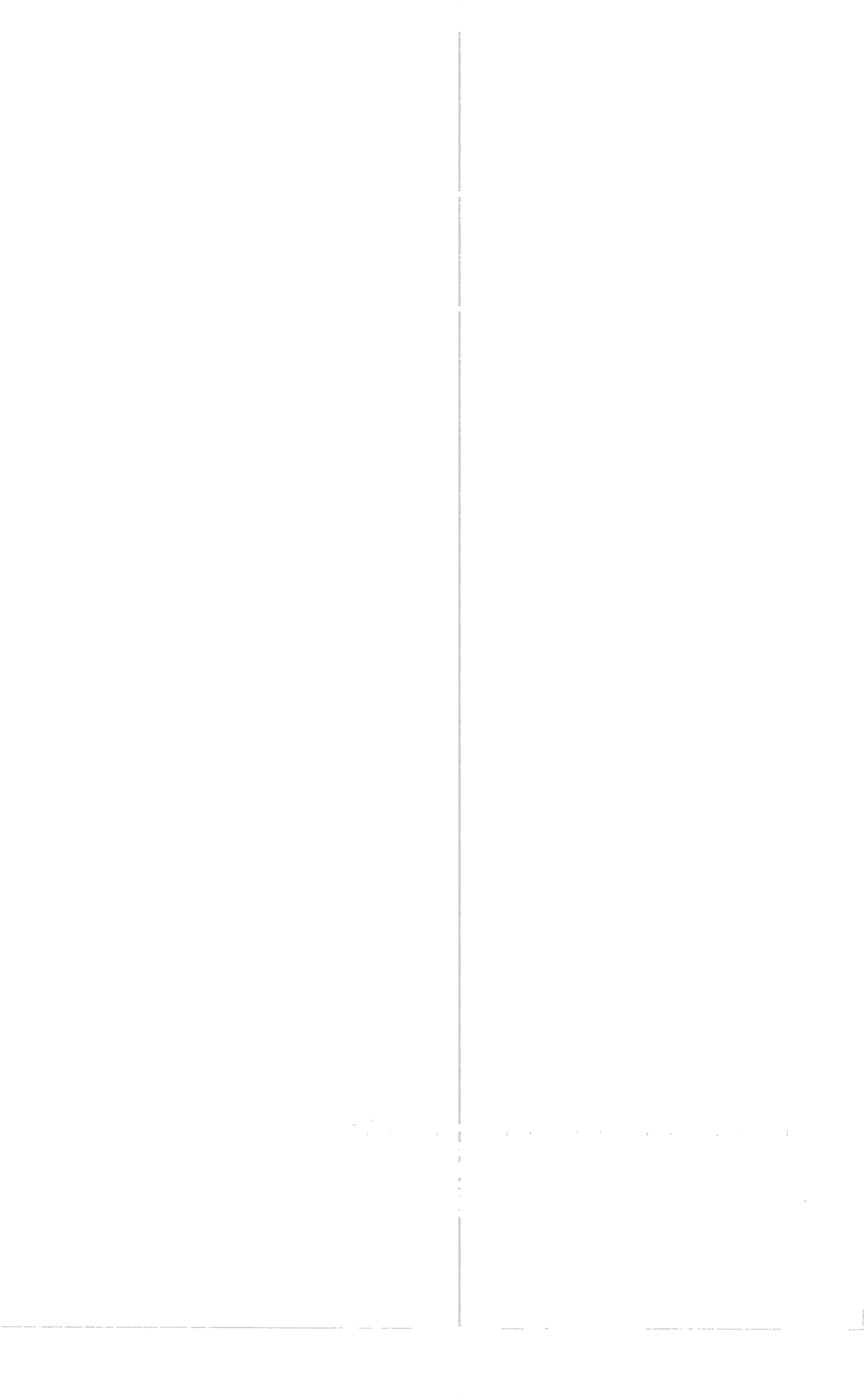

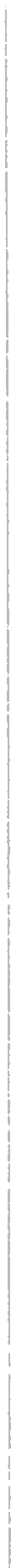

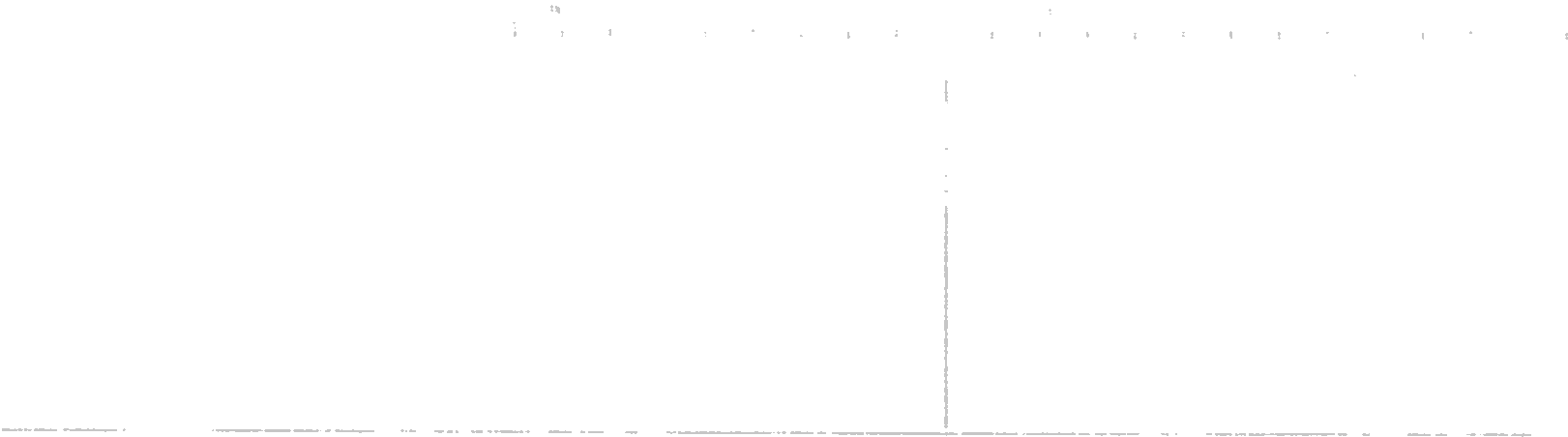

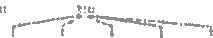

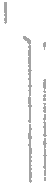

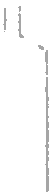

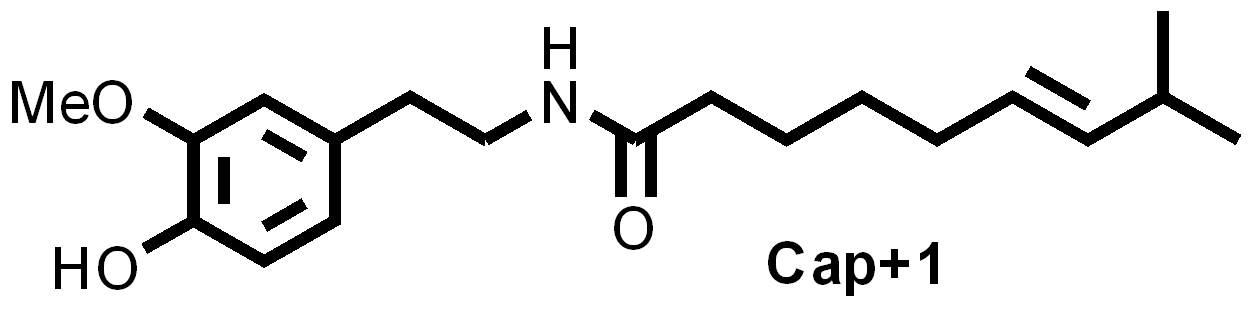

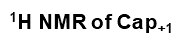

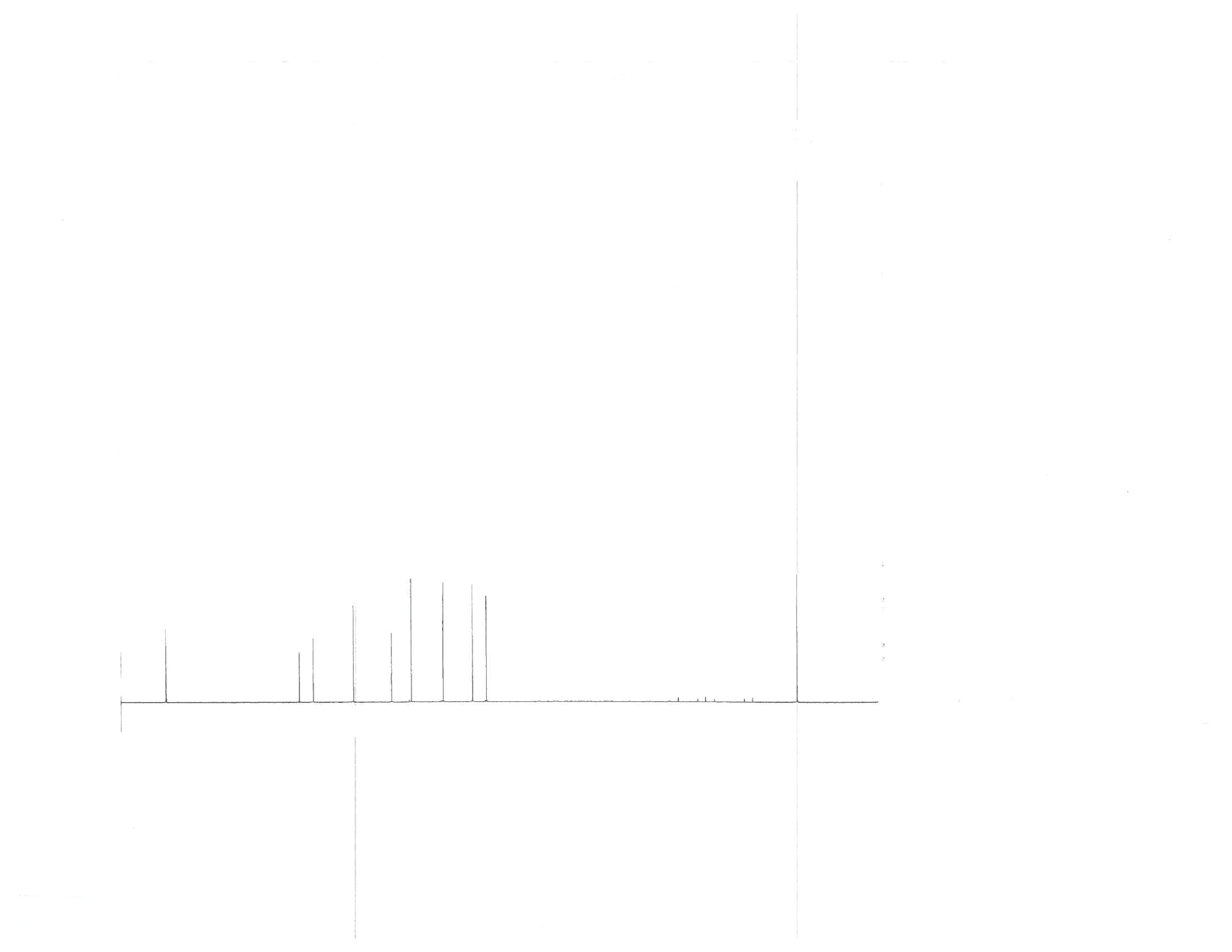

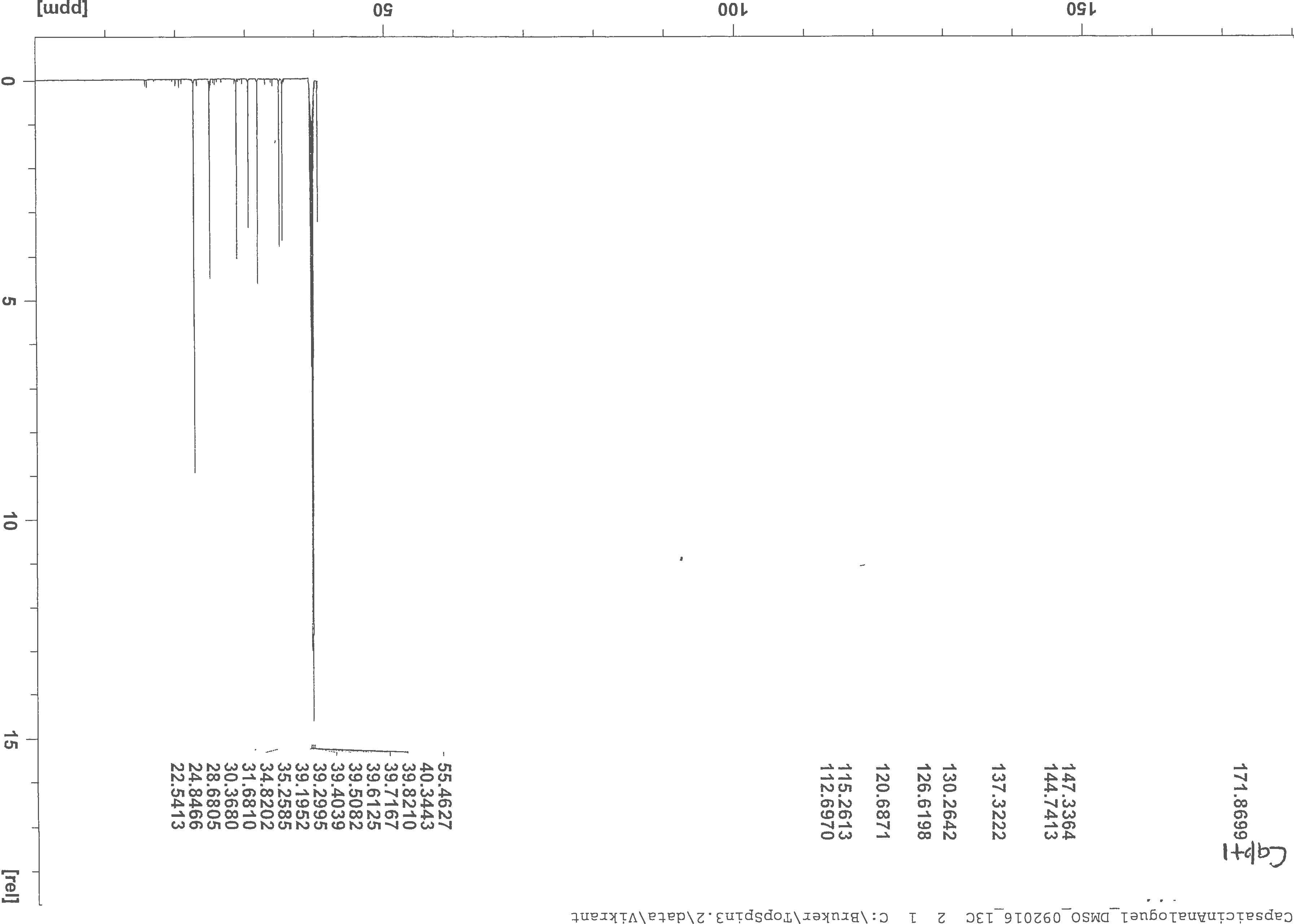

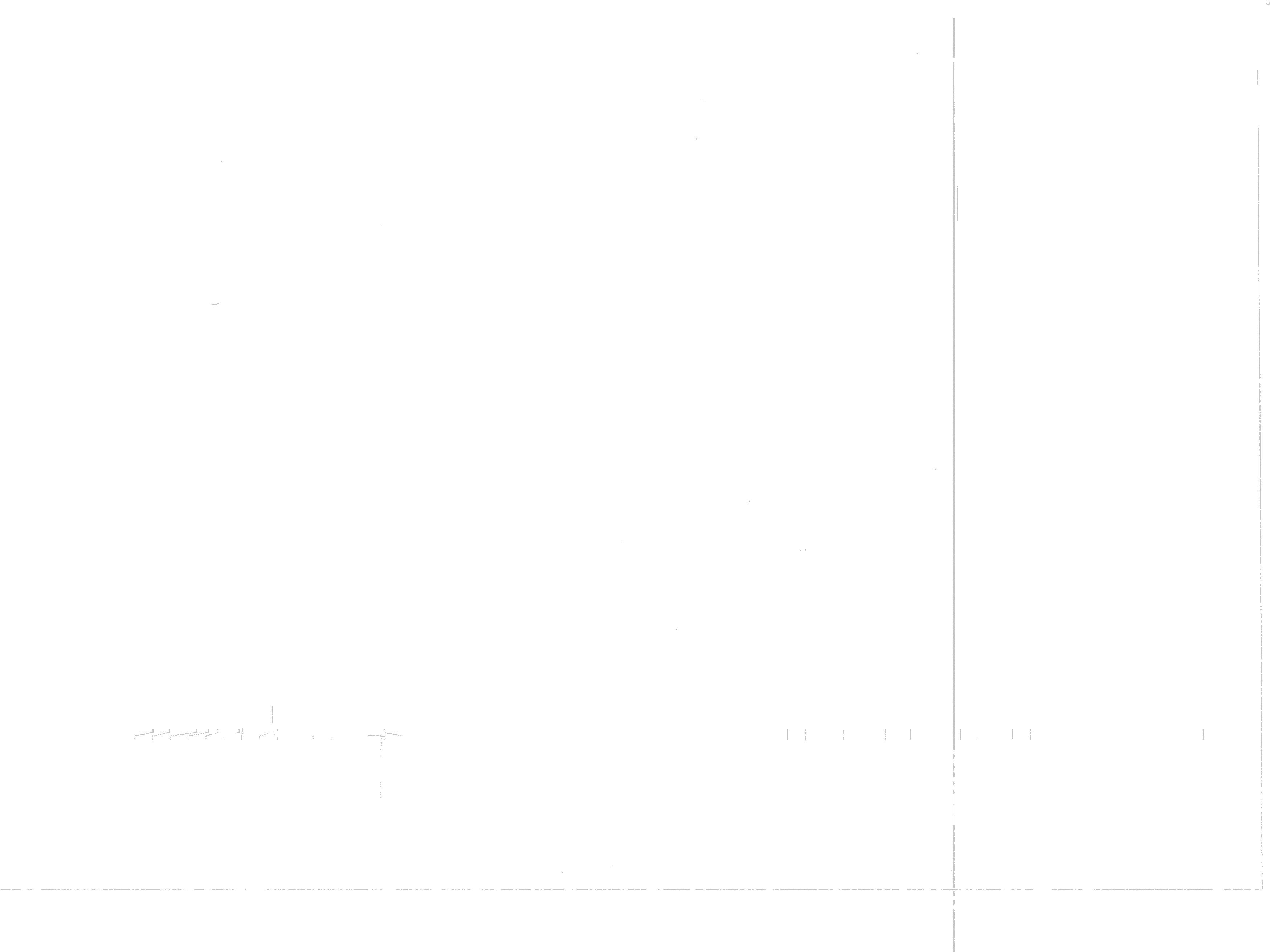

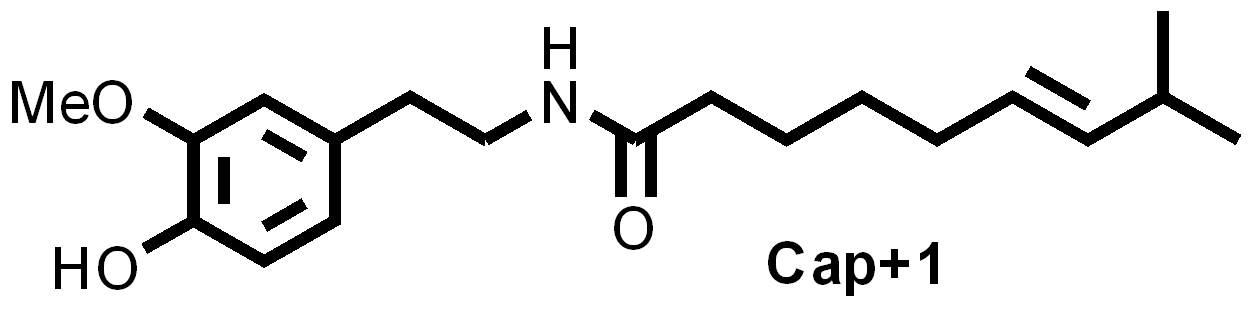

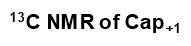

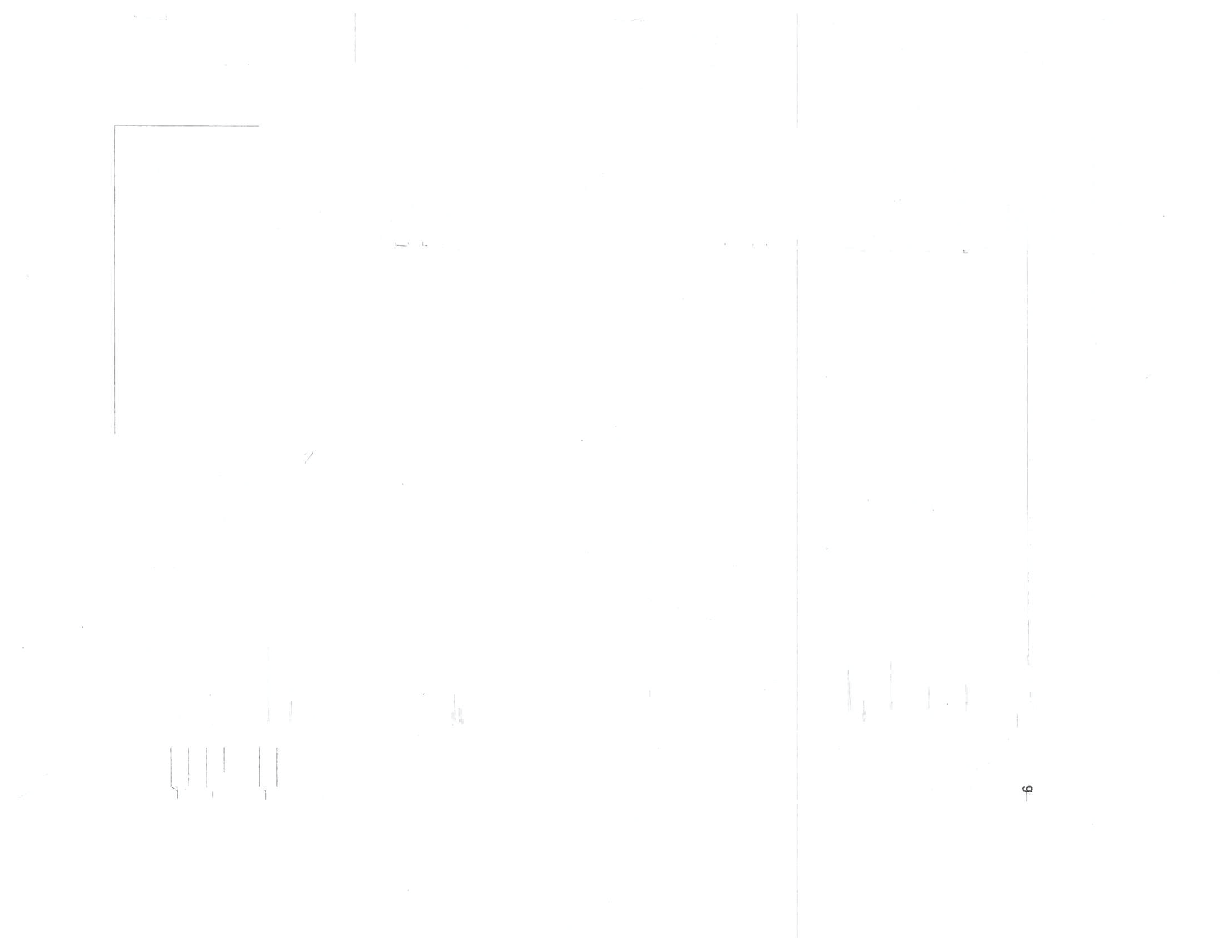

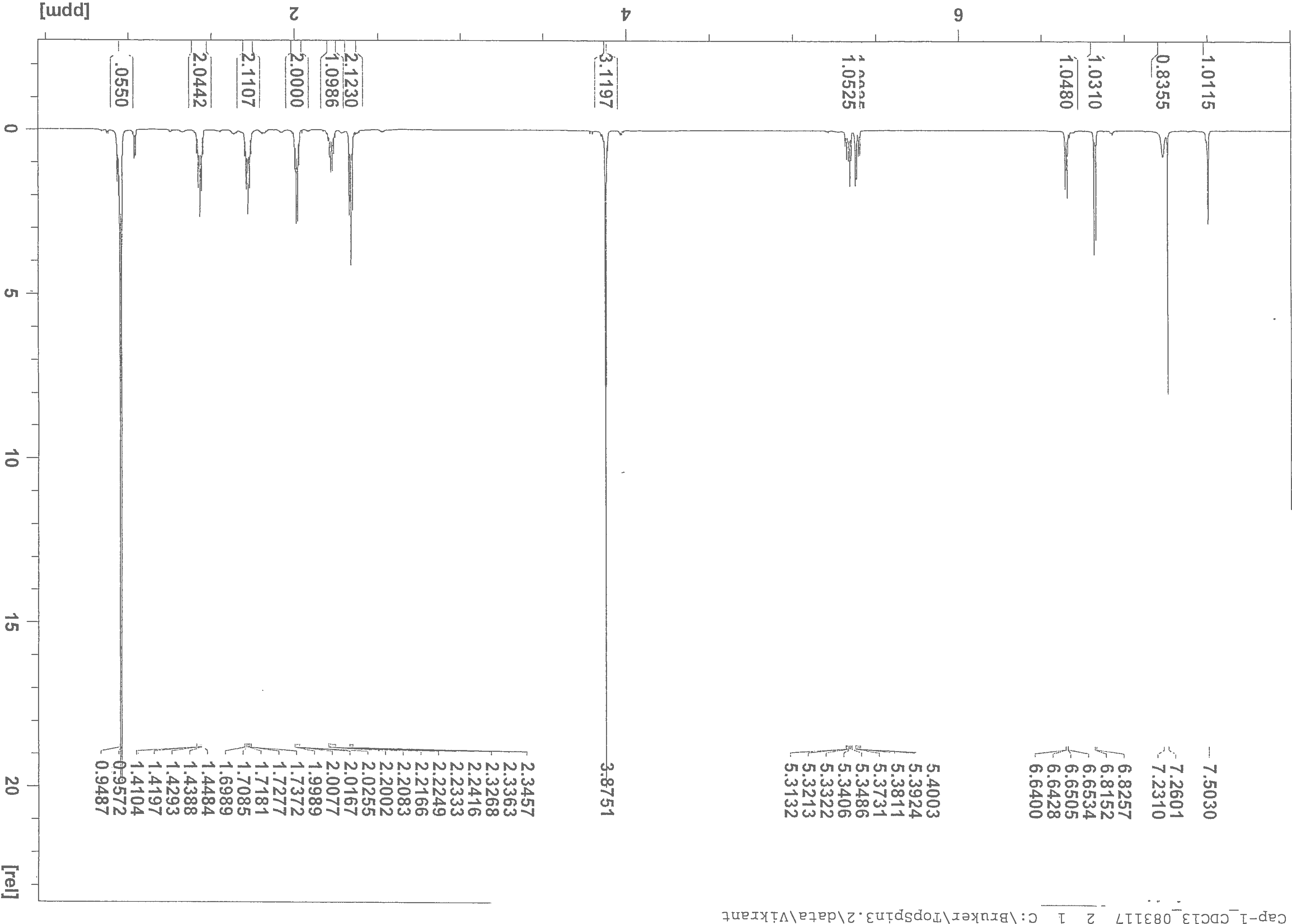

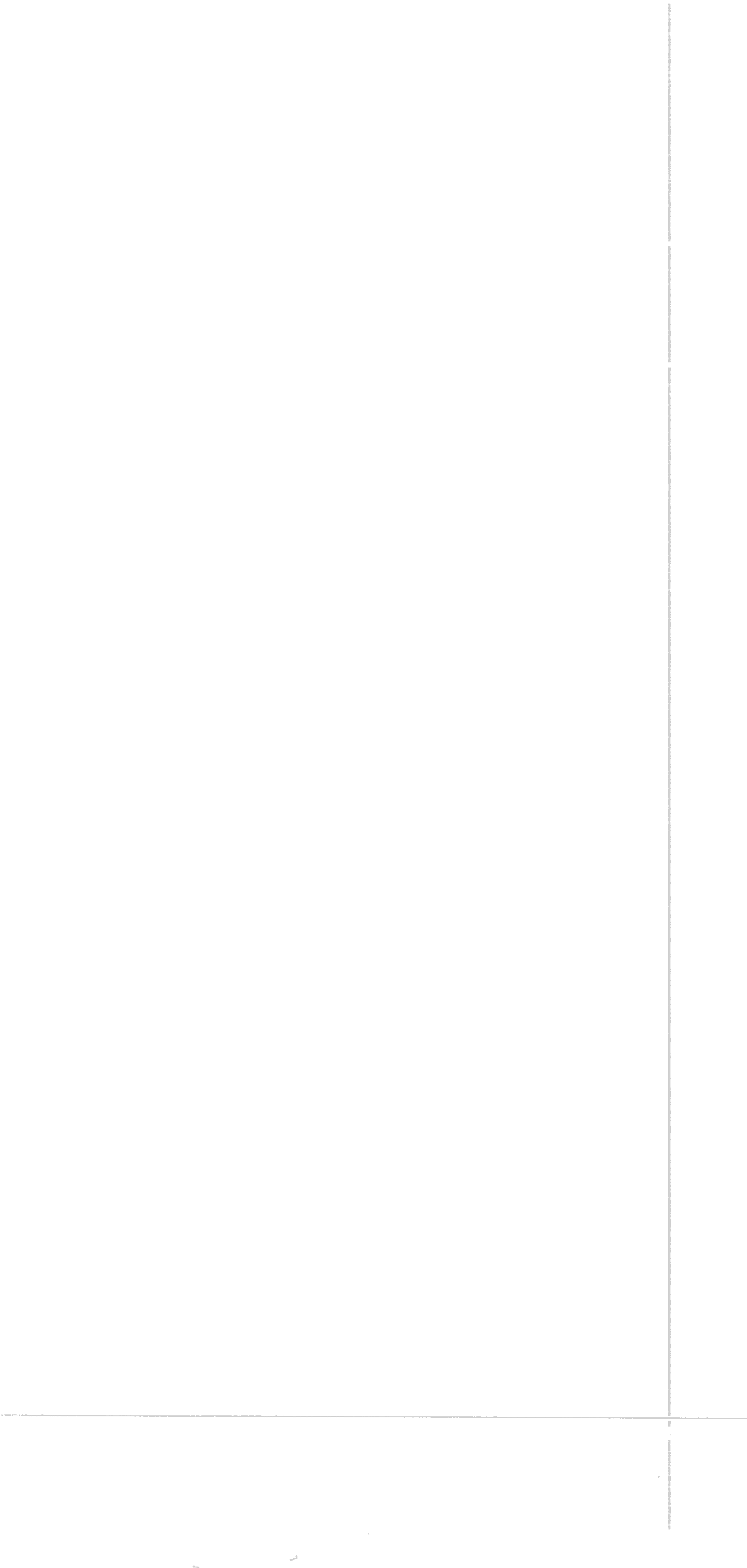

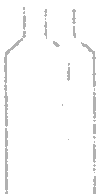

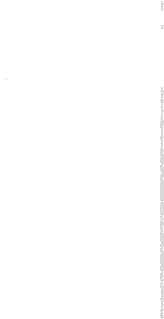

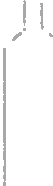

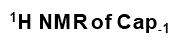

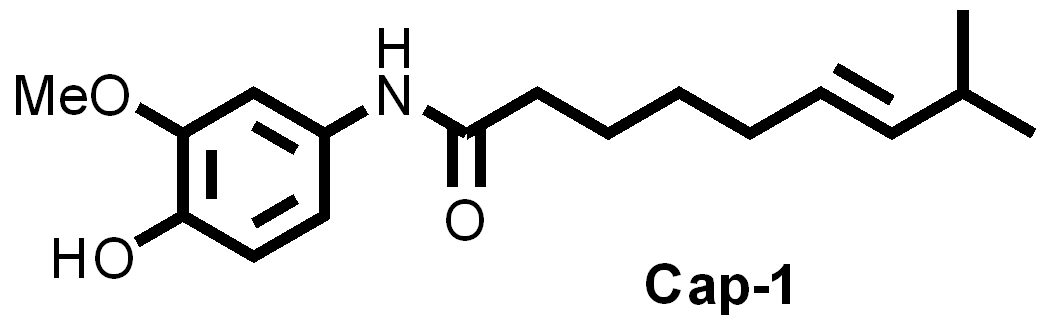

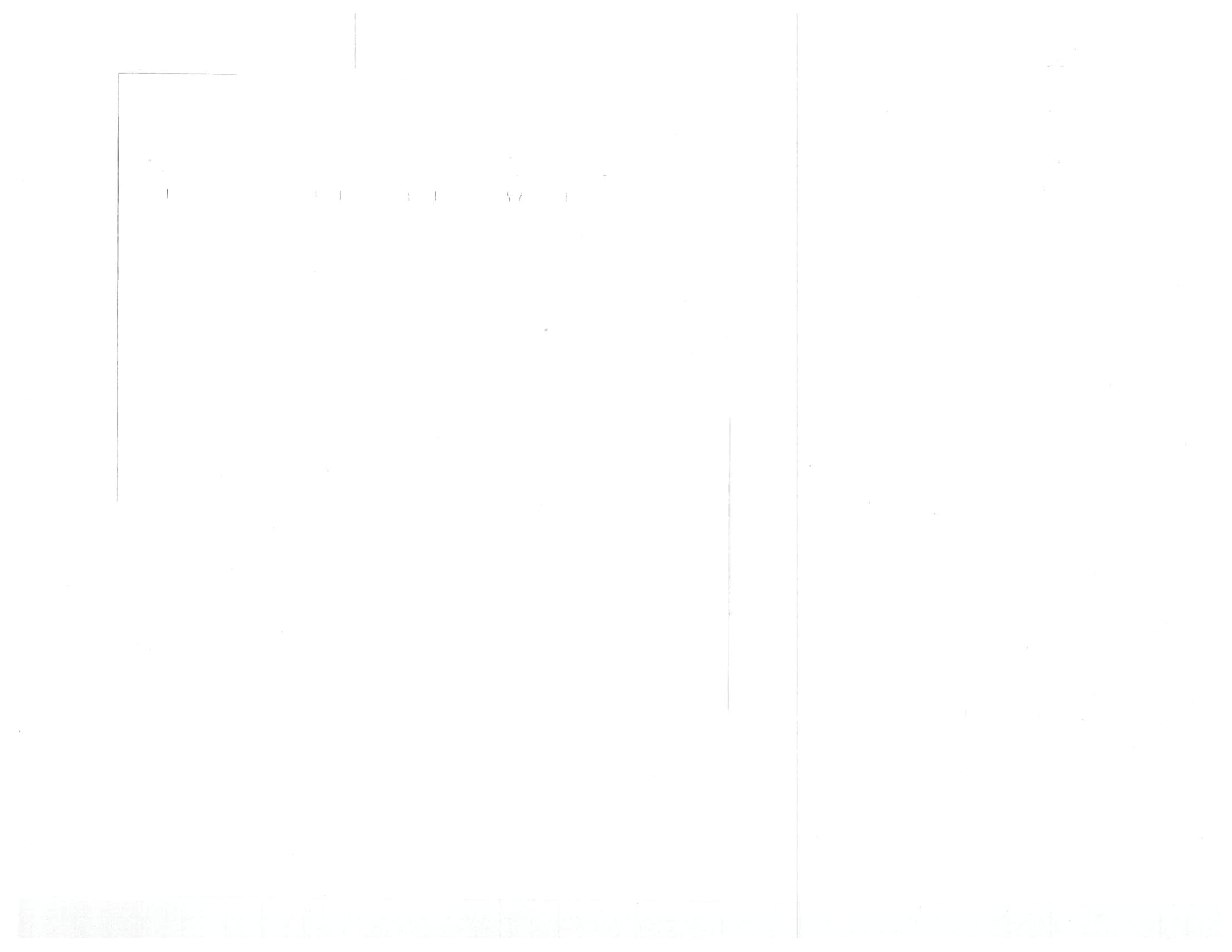

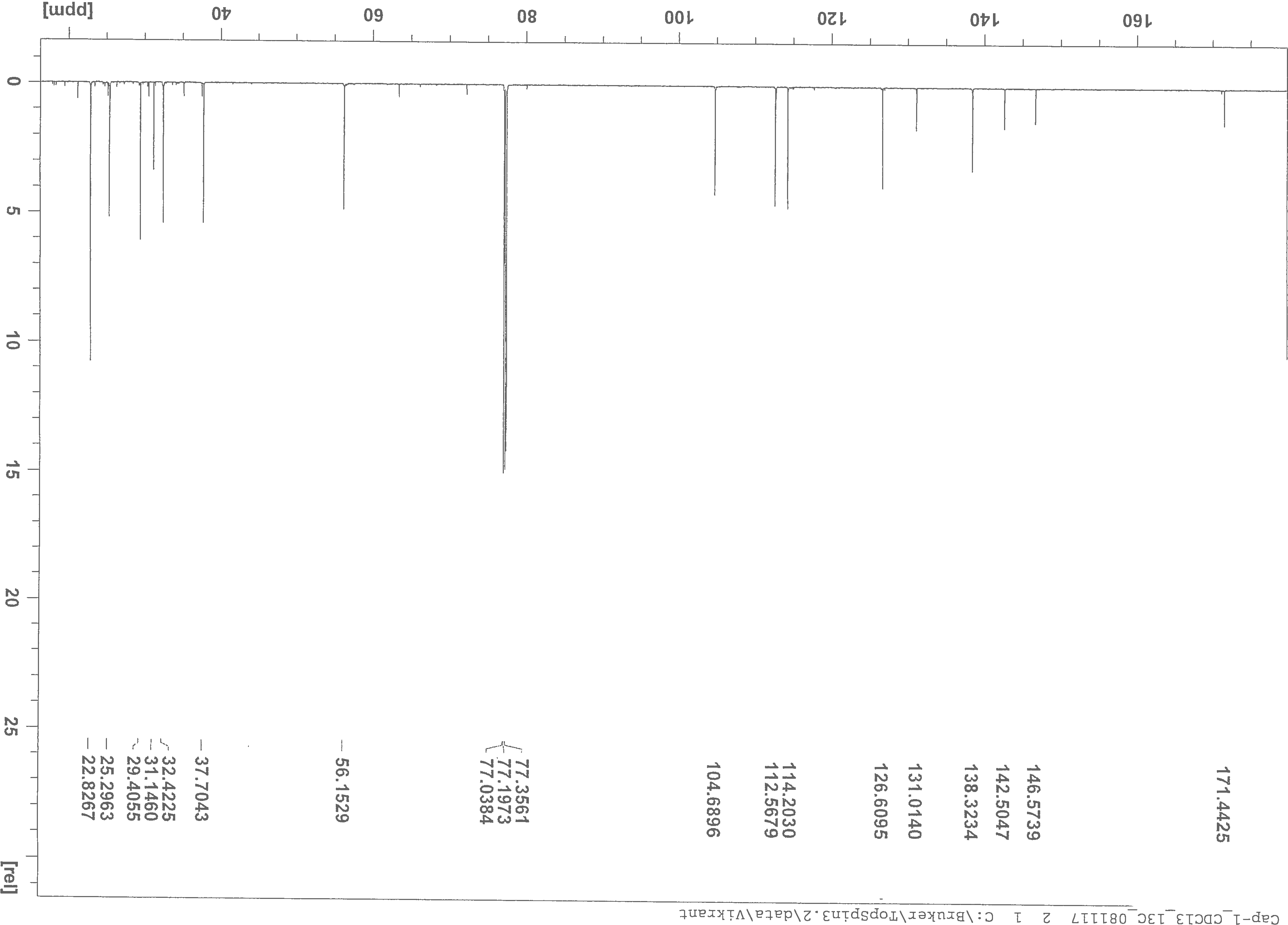

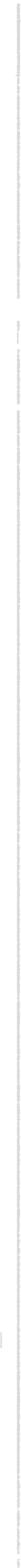

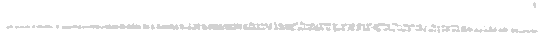

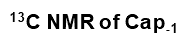

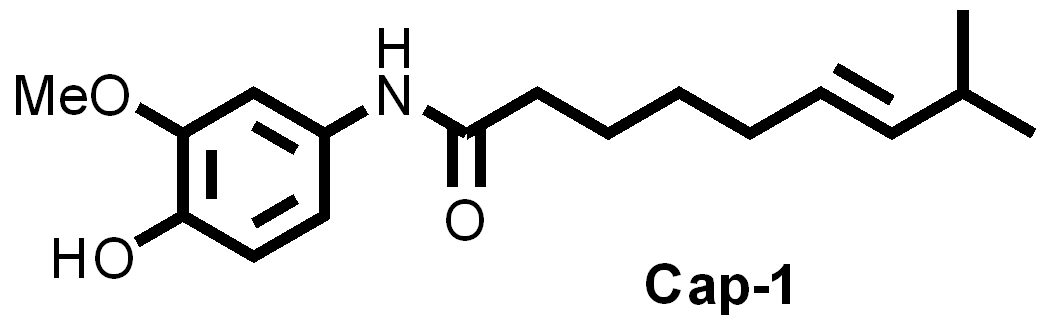

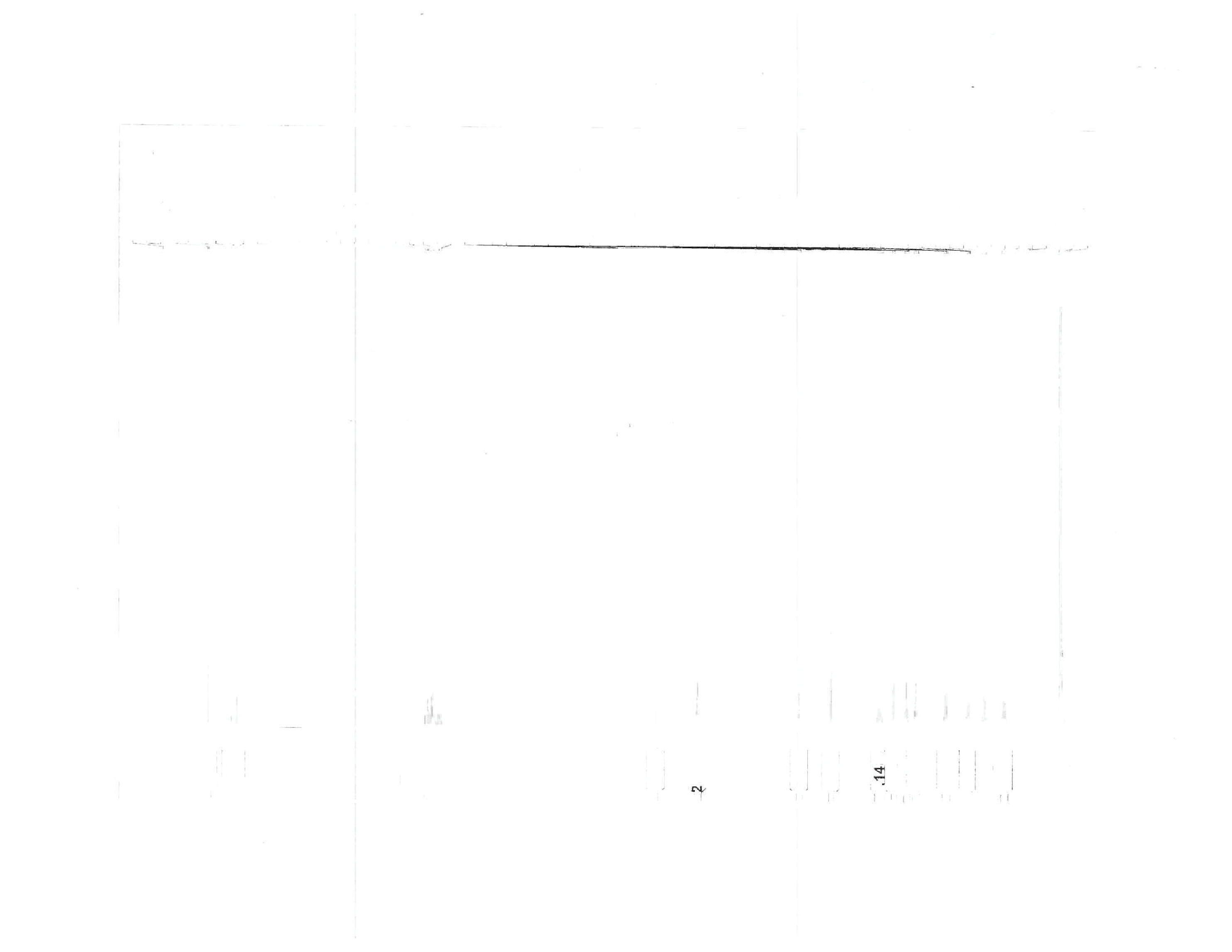

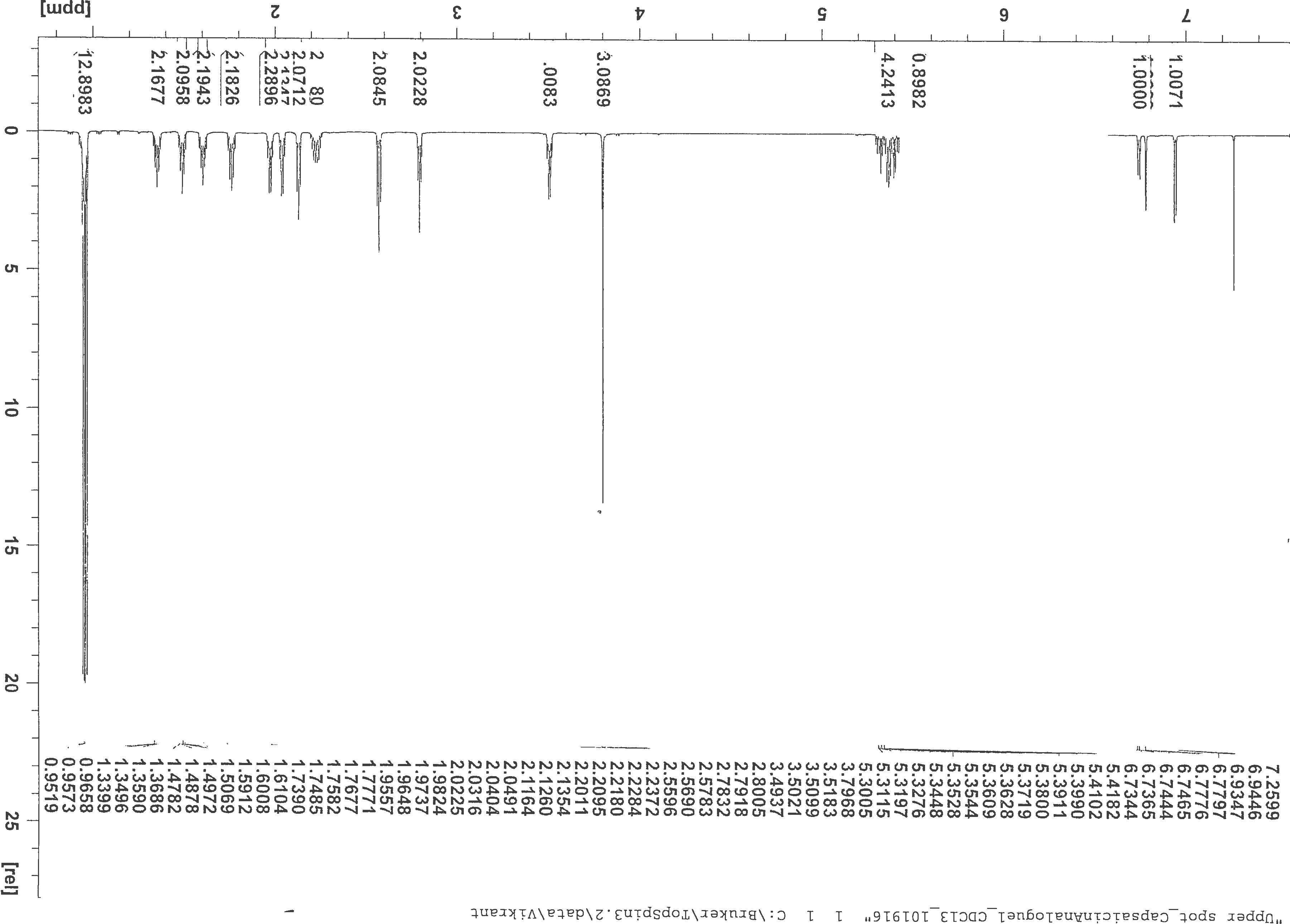

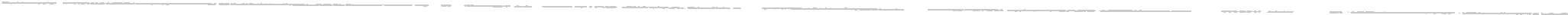

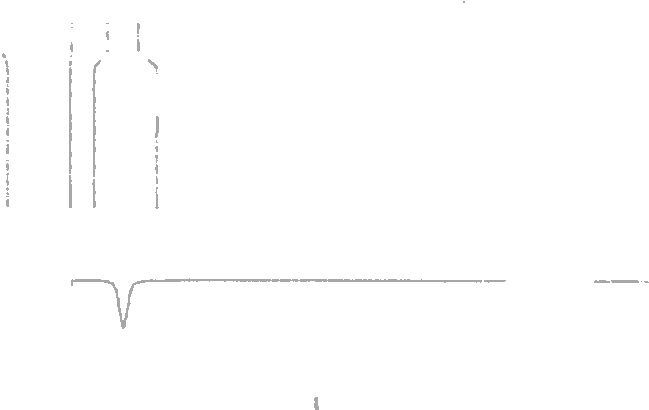

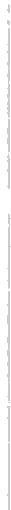

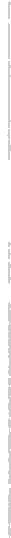

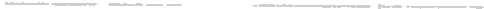

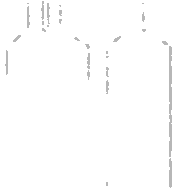

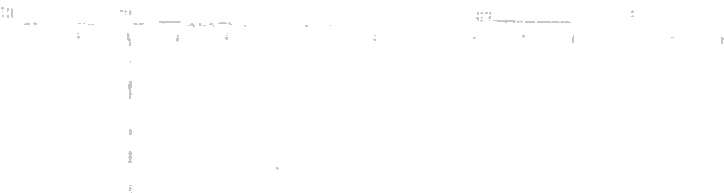

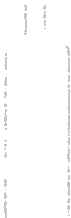

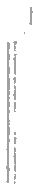

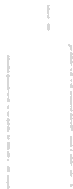

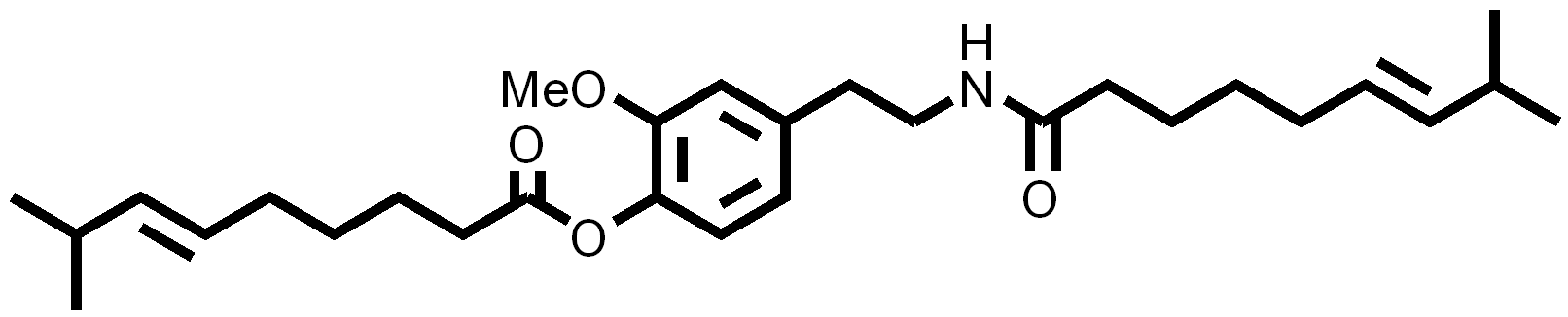

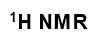

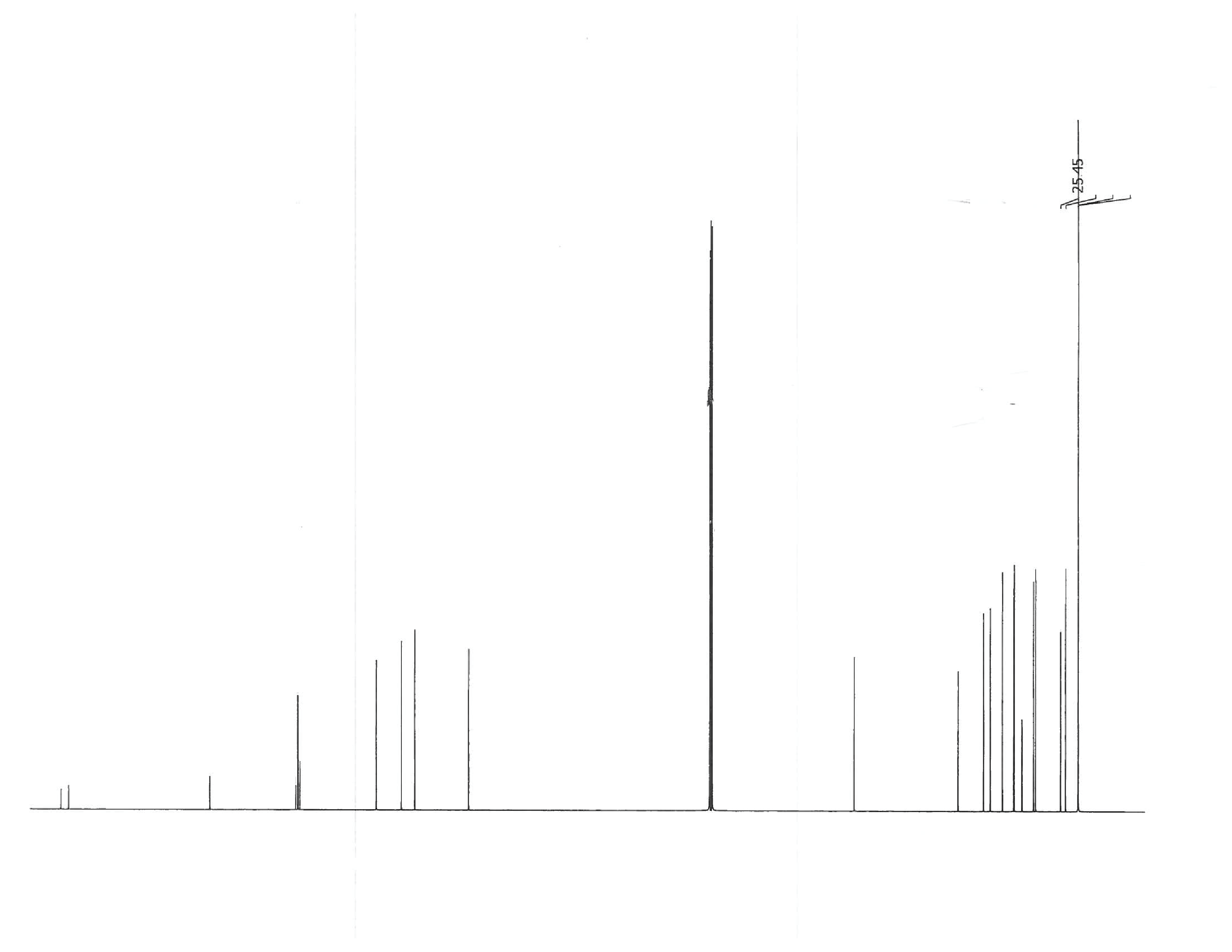

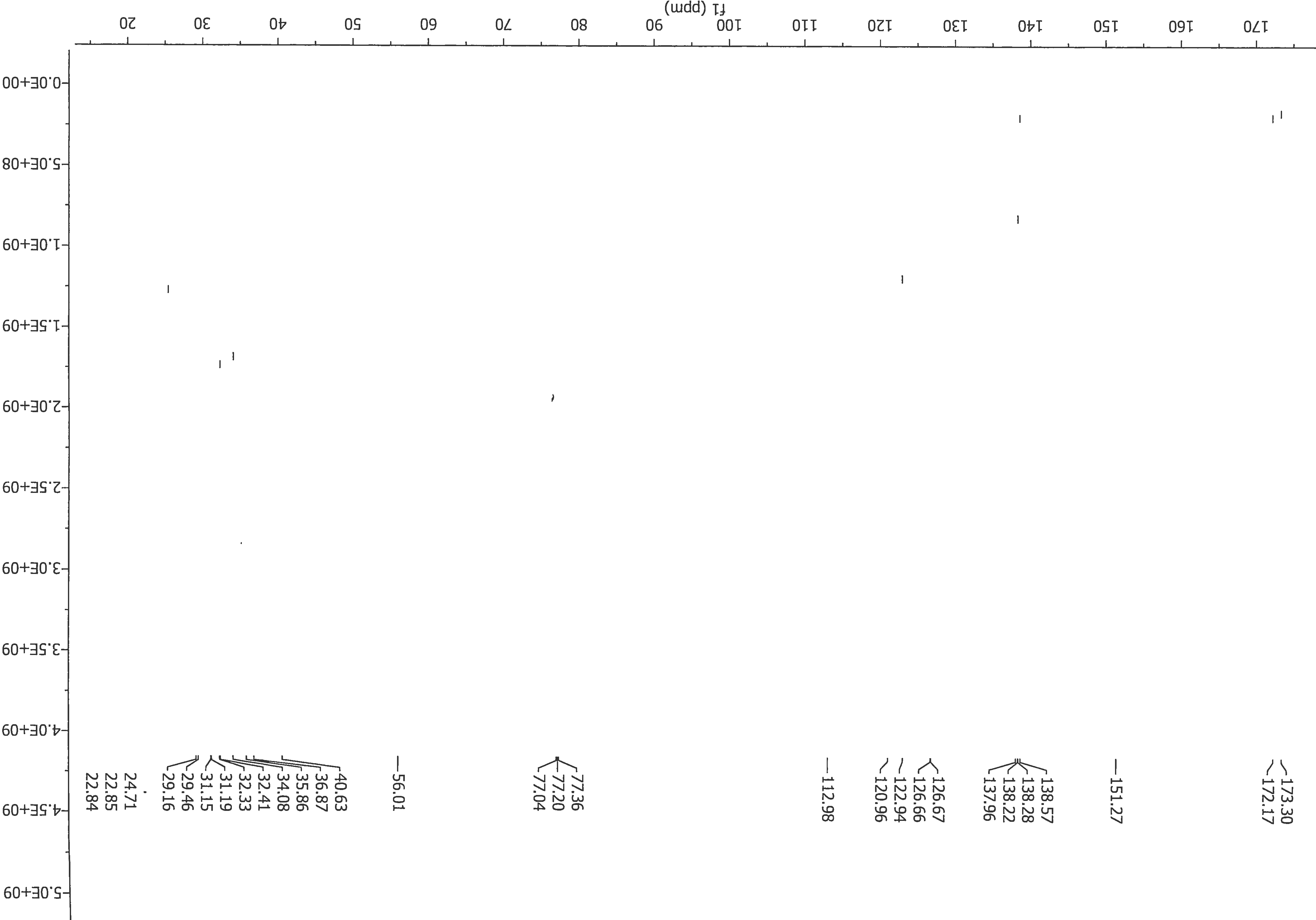

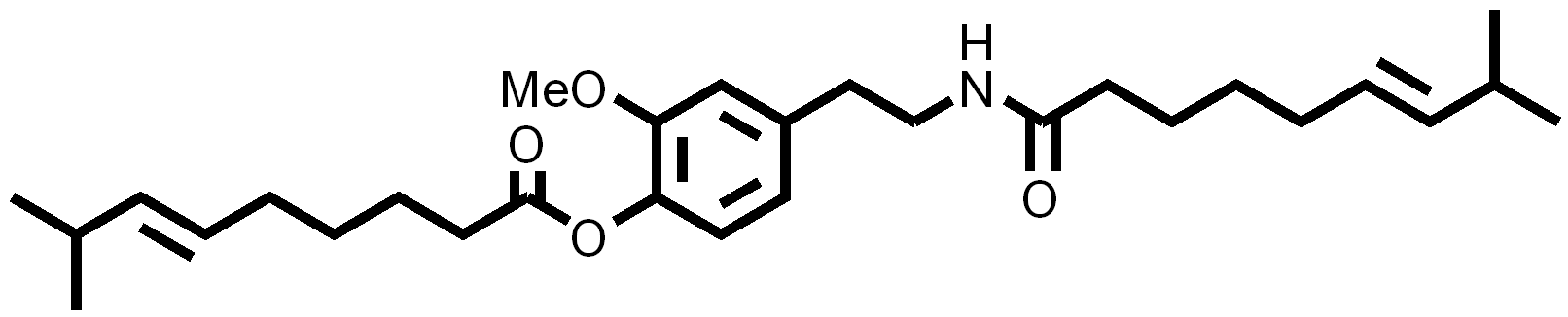

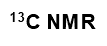


**Commands for Rosetta docking:**

~/rosetta/main/source/bin/rosetta_scripts.linuxgccrelease \

-in:path:database ~/rosetta/main/database \

-in:file:s ~/V1_CAPm1.pdb \

-in:file:extra_res_fa ~/cap-1.fa.params \

-score:weights membrane_highres_Menv_smooth.wts \

-parser:protocol ~/dock-classic-m1.xml \

-packing \

-ex1 \

-ex2 \

-use_input_sc \

-extrachi_cutoff 3 \

-improve_orientation 1000 \

-nstruct 300 \

-out:overwrite \

-out:prefix dock-full- \

-out:file:silent ~/${SLURM_ARRAY_TASK_ID}/dock-full_3J5R_${SLURM_ARRAY_TASK_ID}.silent \

-out:file:silent_struct_type binary \

**Rosetta scripts for capsaicin analogs docking:**

<ROSETTASCRIPTS>

<SCOREFXNS>

<memb_lowres weights="membrane_highres_Menv_smooth" />

<memb_highres weights="membrane_highres_Menv_smooth" />

</SCOREFXNS>

<LIGAND_AREAS>

<docking_sidechain chain="X" cutoff="6.0" add_nbr_radius="true" all_atom_mode="true" minimize_ligand="10"/>

<final_sidechain chain="X" cutoff="6.0" add_nbr_radius="true" all_atom_mode="true"/> <final_backbone chain="X" cutoff="7.0" add_nbr_radius="false" all_atom_mode="true"

Calpha_restraints="5"/>

</LIGAND_AREAS>

<INTERFACE_BUILDERS>

<side_chain_for_docking ligand_areas="docking_sidechain"/>

<side_chain_for_final ligand_areas="final_sidechain"/>

<backbone ligand_areas="final_backbone" extension_window="3"/>

</INTERFACE_BUILDERS>

<MOVEMAP_BUILDERS>

<docking sc_interface="side_chain_for_docking" minimize_water="true"/>

<final sc_interface="side_chain_for_final" bb_interface="backbone" minimize_water="true"/>

</MOVEMAP_BUILDERS>

<SCORINGGRIDS ligand_chain="X" width="15">

<vdw grid_type="ClassicGrid" weight="1.0"/>

</SCORINGGRIDS>

<MOVERS>

<MembraneTopology name="topology" span_file="~/V1TM_3J5P.span"/>

<Transform name="transform" chain="X" box_size="5.0" move_distance="0.5" angle="5" cycles="1000" repeats="1" temperature="5" initial_perturb="5.0"/>

<HighResDocker name="high_res_docker" cycles="6" repack_every_Nth="3" scorefxn="memb_lowres" movemap_builder="docking"/>

<FinalMinimizer name="final" scorefxn="memb_highres" movemap_builder="final"/>

<InterfaceScoreCalculator name="add_scores" chains="X" scorefxn="memb_highres"/>

<ParsedProtocol name="low_res_dock">

<Add mover_name="transform"/>

</ParsedProtocol>

<ParsedProtocol name="high_res_dock">

<Add mover_name="high_res_docker"/>

<Add mover_name="final"/>

</ParsedProtocol>

<ParsedProtocol name="reporting">

<Add mover_name="add_scores"/>

</ParsedProtocol>

</MOVERS>

<PROTOCOLS>

<Add mover="topology"/>

<Add mover_name="low_res_dock"/>

<Add mover_name="high_res_dock"/>

<Add mover_name="reporting"/>

</PROTOCOLS>

</ROSETTASCRIPTS>
